# Novel biomarker-based score (SAD-60) for predicting mortality in patients with COVID-19 pneumonia: a multicenter retrospective cohort of 1013 patients

**DOI:** 10.2217/bmm-2021-1085

**Published:** 2022-03-30

**Authors:** Serkan Surme, Gulsah Tuncer, Osman F Bayramlar, Betul Copur, Esra Zerdali, Inci Y Nakir, Meltem Yazla, Ahmet Buyukyazgan, Ayse RK Cinar, Yesim Kurekci, Mustafa Alkan, Yusuf E Ozdemir, Gonul Sengoz, Filiz Pehlivanoglu

**Affiliations:** ^1^Department of Infectious Diseases & Clinical Microbiology, Haseki Training & Research Hospital, Istanbul, 34096, Turkey; ^2^Department of Medical Microbiology, Institute of Graduate Studies, Istanbul University-Cerrahpasa, Istanbul, 34098, Turkey; ^3^Department of Public Health, Bakirkoy District Health Directorate, Istanbul, 34140, Turkey; ^4^Department of Infectious Diseases & Clinical Microbiology, Bahcelievler State Hospital, Istanbul, 34186, Turkey; ^5^Department of Infectious Diseases & Clinical Microbiology, Bayrampasa State Hospital, Istanbul, 34040, Turkey; ^6^Department of Infectious Diseases & Clinical Microbiology, Arnavutkoy State Hospital, Istanbul, 34275, Turkey; ^7^Department of Infectious Diseases & Clinical Microbiology, Gaziosmanpasa Training & Research Hospital, Istanbul, 34255, Turkey; ^8^Department of Infectious Diseases & Clinical Microbiology, Bakirkoy Sadi Konuk Training & Research Hospital, Istanbul, 34147, Turkey

**Keywords:** albumin, COVID-19, D-dimer, mortality, SAD-60 score

## Abstract

**Background:** The aim was to explore a novel risk score to predict mortality in hospitalized patients with COVID-19 pneumonia. **Methods:** This was a retrospective, multicenter study. **Results:** A total of 1013 patients with COVID-19 were included. The mean age was 60.5 ± 14.4 years, and 581 (57.4%) patients were male. In-hospital death occurred in 124 (12.2%) patients. Multivariate analysis revealed peripheral capillary oxygen saturation (SpO2), albumin, D-dimer and age as independent predictors. The mortality score model was given the acronym SAD-60, representing **S**pO2, **A**lbumin, **D**-dimer, age **≥60** years. The SAD-60 score (0.776) had the highest area under the curve compared with CURB-65 (0.753), NEWS2 (0.686) and qSOFA (0.628) scores. **Conclusion:** The SAD-60 score has a promising predictive capacity for mortality in hospitalized patients with COVID-19.

The pandemic of COVID-19 continues to be a significant public health issue [1]. COVID-19 has caused hospitalization and intensive care unit (ICU) admission, and results in mortality especially in older adults with comorbid diseases [2]. Previous studies have identified the epidemiological features, clinical characteristics and outcomes of COVID-19 [2–8]. Wu *et al.* reported that 14% of the 72,314 patients had a severe illness, and the mortality rate among 44,672 critical patients with confirmed COVID-19 was 49.0% [2]. Mortality rates in critically ill patients were varied and reported to be as high as 61.5% [2,4,9].

Estimating the risk of poor prognosis and early recognition of critical disease in patients with COVID-19 is important for the care planning process in reducing morbidity and mortality, as immediate intensive care treatment is a crucial step to recovery [9]. Patient outcomes would be improved by prioritizing patient care resources especially in limited-resource settings [10]. Several studies have determined predictors for clinical deterioration and mortality [11–13]. Moreover, recent studies have focused on the discriminative ability of previously known risk scores for community-acquired pneumonia in patients with COVID-19 [14–17]. Further, prediction models for early identification of COVID-19 progression have been reported [18–22]. However, although recent studies have created various novel scores, there is no certain and specific risk score to predict poor prognosis in patients with COVID-19.

In this study, the aim was to explore a novel risk score to predict mortality in hospitalized patients with COVID-19 pneumonia. In addition, the accuracy of the novel risk score was compared with CURB-65, qSOFA and NEWS2 scores.

## Patients & methods

This retrospective multicenter study was conducted in all hospitalized adult patients (≥18 years old) with laboratory and radiologically confirmed COVID-19 pneumonia who were diagnosed by the Department of Infectious Diseases and Clinical Microbiology between 1 November 2020 and 30 November 2020 from six public/governmental hospitals in Istanbul, Turkey. Demographic features, clinical findings, laboratory results and patient outcomes from medical records via a datasheet form were retrospectively collected. Peripheral capillary oxygen saturation (SpO2) was measured on room air. All data was recorded at admission or within 24 h after hospitalization. SARS-CoV-2 real-time reverse transcription PCR (RT-PCR) was performed in samples collected by nasopharyngeal and/or oropharyngeal swabs. Chest computed tomography confirmed patients with COVID-19 pneumonia requiring hospitalization were included, whereas asymptomatic patients, outpatients and radiologically unconfirmed patients were excluded.

A confirmed COVID-19 pneumonia was defined as a case diagnosed with a molecularly and radiologically confirmed COVID-19 pneumonia by both SARS-CoV-2 RT-PCR and chest computed tomography among suspected patients. Underlying diseases including hypertension, diabetes mellitus, congestive heart failure, chronic artery disease, chronic renal failure and cerebrovascular disease, which were significantly more frequent in deceased patients, were defined as a new variable named ‘any comorbidity’. Mortality was defined as all-cause in-hospital death.

The primary outcome of this study was in-hospital death. Secondary outcome was ICU admission or in-hospital death. Once the novel score was developed, the predictive ability of the novel score for both outcomes was compared with CURB-65, NEWS2 and qSOFA.

Continuous variables were described as median ± interquartile range, whereas categorical variables were described as numbers and percentages. Chi-square and Fisher’s exact tests were performed to compare categorical variables. The independent sample t-test was performed for variables with normal distribution, whereas the Mann–Whitney *U* test was performed for variables without normal distribution. The optimal cut-off values of the independent variables were calculated by Youden’s index of the receiver operating characteristic (ROC) curve. ROC analyses defined cut-off values for candidate variables were performed before univariate and multivariate analyses regarding survival. Univariate analysis was performed, and all significant variables except parameters with high percentages (>20%) of missing values were included in multivariable logistic regressions. Odds ratio (OR) values with 95% CI were calculated. Independent predictors were identified using multivariate logistic regression analysis. When there was a strong correlation, only the variable with a high contribution to the model was included. OR values of the variables in a nomogram were used to assign score points. Score points were rounded to the nearest 0.5 or whole number, whichever was closer, to make the calculation easier. In addition, ROC analysis with area under the curve (AUC) was used to evaluate the performance of the novel score. Statistical Package for Social Sciences (SPSS) version 20.0 was used for statistical analyses. A p-value <0.05 was considered statistically significant.

All procedures were performed in accordance with the ethical standards of the Declaration of Helsinki and National Research Committee. This study was approved by the Ethics Committee of Haseki Training and Research Hospital (approval number: 2020–239; date: 23 December 2020). Written informed consent was waived given the retrospective nature of this study.

## Results

A total of 1013 patients with COVID-19 were included. The median age was 61 ± 21 years, and 581 (57.4%) patients were male. A total of 672 patients (66.3%) had at least one comorbidity. Increased age was significantly associated with mortality (p < 0.001). The most common comorbidities were hypertension (n = 428, 42.3%) and diabetes mellitus (n = 323, 31.9%). Diabetes mellitus (41.1% vs 30.6%, p = 0.02), hypertension (58.1% vs 40.0%, p < 0.001), congestive heart failure (16.1% vs 4.3%, p = 0.04), chronic artery disease (21.8% vs 13.0%, p = 0.01), chronic renal failure (16.1% vs 4.3%, p < 0.001) and cerebrovascular disease (6.5% vs 2.3%, p = 0.01) were more frequent in deceased patients compared with survived patients. The median length of hospital stay was 9 ± 7 days. The median duration from hospitalization to death was 13 ± 11 days. A total of 331patients (32.7%) had severe or critical COVID-19 on admission defined by WHO criteria. A total of 175 (17.3%) patients were admitted to the ICU. In-hospital death occurred in 124 (12.2%) patients. A total of 18 patients (14.5%) died during the first week of hospitalization. Demographic characteristics of survived and deceased patients with COVID-19 are available in Table 1.

**Table 1.** Demographic characteristics and clinical outcomes of patients with COVID-19.

Parameters	Mortality			
	No	Yes	Total	p-value
	n (%)	n (%)	n (%)	
**Age (years)**				**<0.001**
– IQR	21	17	21	
– Median	59	71	61	
**Age group (years)**				
– 18–64	574 (64.6)	42 (33.9)	616 (60.8)	**<0.001**
– 65–74	191 (21.5)	37 (29.8)	228 (22.5)	
– 75–84	98 (11)	27 (21.8)	125 (12.3)	
– >84	26 (2.9)	18 (14.5)	44 (4.3)	
**Gender**				
– Male	516 (58)	65 (52.4)	581 (57.4)	0.24
– Female	373 (42)	59 (47.6)	432 (42.6)	
**Underlying diseases**	569 (64)	103 (83)	672 (66.3)	**<0.001**
– COPD	20 (2.3)	4 (3.2)	24 (2.4)	0.52
– Diabetes mellitus	272 (30.6)	51 (41.1)	323 (31.9)	**0.02**
– Hypertension	356 (40.0)	72 (58.1)	428 (42.3)	**<0.001**
– Congestive heart failure	36 (4.0)	10 (8.1)	46 (4.5)	**0.04**
– Chronic artery disease	116 (13.0)	27 (21.8)	143 (14.1)	**0.01**
– Chronic renal failure	38 (4.3)	20 (16.1)	58 (5.7)	**<0.001**
– Malignancy	21 (2.4)	6 (4.8)	27 (2.7)	0.11
– Cerebrovascular disease	20 (2.3)	8 (6.5)	28 (2.8)	**0.01**
– Rheumatological disease	18 (2)	5 (4)	23 (2.3)	0.16
– Neurological disorder	16 (1.8)	5 (4)	21 (2)	0.10
**Clinical outcomes**				
– Invasive ventilation	19 (2.1)	116 (93.6)	135 (13.3)	**<0.001**
– ICU admission	57 (6.4)	118 (95.2)	175 (17.3)	**<0.001**
– Dialysis	1 (0.1)	10 (8.1)	11 (1.1)	**<0.001**
– Noninvasive ventilation	57 (6.4)	115 (92.7)	172 (17)	**<0.001**

COPD: Chronic obstructive pulmonary disease; ICU: Intensive care unit; IQR: Interquartile range.

The most common symptoms in the cohort were dyspnea (n = 651, 64.3%), cough (n = 581, 57.4%), fatigue (n = 505, 49.9%) and fever (n = 274, 27.0%). Dyspnea (72.6% vs 63.1%, p = 0.04), vomiting (16.1% vs 8.2%, p = 0.01), diarrhea (9.7% vs 5.3%, p = 0.05) and confusion (5.3% vs 0.7%, p < 0.001) were more frequent in deceased patients compared with survived patients. Median respiratory rate (24 ± 8 vs 20 ± 4, p < 0.001) and SpO2 on room air (88 ± 10 vs 92 ± 6, p < 0.001) were higher in deceased patients than survived patients. Comparison of clinical presentations of survived and deceased patients with COVID-19 are represented in Table 2.

**Table 2.** Clinical presentations of patients with COVID-19.

Symptoms	Mortality			
	No	Yes	Total	p-value
	n (%)	n (%)	n (%)	
Fever	249 (28)	25 (20.2)	274 (27)	0.07
Cough	514 (57.8)	67 (54)	581 (57.4)	0.43
Dyspnea	561 (63.1)	90 (72.6)	651 (64.3)	**0.04**
Chest pain	44 (5)	3 (2.4)	47 (4.6)	0.21
Myalgia	164 (18.5)	24 (19.4)	188 (18.6)	0.81
Arthralgia	114 (12.8)	20 (16.1)	134 (13.2)	0.31
Fatigue	435 (48.9)	70 (56.5)	505 (49.9)	0.12
Sore throat	26 (2.9)	7 (5.7)	33 (3.3)	0.11
Abdominal pain	32 (3.6)	8 (6.5)	40 (4)	0.13
Nausea	133 (15)	23 (18.6)	156 (15.4)	0.30
Vomiting	73 (8.2)	20 (16.1)	93 (9.2)	**0.01**
Diarrhea	47 (5.3)	12 (9.7)	59 (5.8)	**0.05**
Anosmia	46 (5.2)	8 (6.5)	54 (5.3)	0.55
Ageusia	45 (5.1)	7 (5.7)	52 (5.1)	0.78
Confusion	6 (0.7)	5 (4.3)	11 (1.1)	**0.001**

**Vital signs**	**Median (IQR)**	**Median (IQR)**	**Median (IQR)**	**p-value**
Heart rate/min	86 (18)	86 (22)	86 (18)	0.83
SpO2 (%)	92 (6)	88 (10)	92 (7)	**<0.001**
Body temperature (°C)	36.6 (0.8)	36.6 (0.9)	36.6 (0.8)	0.66
Systolic blood pressure (mmHg)	120 (20)	120 (24)	120 (20)	0.60
Diastolic blood pressure (mmHg)	70 (10)	70 (20)	70 (10)	0.44
Respiratory rate/min	20 (4)	24 (8)	20 (6)	**<0.001**

IQR: Interquartile range; SpO2: Peripheral capillary oxygen saturation.

Median neutrophil count (5725 ± 4410 vs 4870 ± 3560, p < 0.001), neutrophil to lymphocyte ratio (4.31 ± 5.97 vs 3.89 ± 4.15, p < 0.001), C-reactive protein (CRP) (104 ± 102 vs 81 ± 87, p < 0.001), procalcitonin (0.3 ± 0.5 vs 0.1 ± 0.1, p < 0.001), D-dimer (1.3 ± 2.1 vs 0.8 ± 0.9, p < 0.001), creatinine (1.2 ± 0.8 vs 0.9 ± 0.4, p < 0.001), urea (51 ± 46 vs 32 ± 19, p < 0.001), lactate dehyrogenase (393 ± 226 vs 338 ± 167, p < 0.001) and urea to albumin ratio (1.47 ± 1.90 vs 0.87 ± 0.59, p < 0.001) were significantly higher in patients who died than those who survived. Median lymphocyte count (920 ± 680 vs 1170 ± 750, p < 0.001), platelet count (179 ± 100 vs 206 ± 109, p = 0.01), glomerular filtration rate (56 ± 49 vs 85 ± 34, p < 0.001) and albumin (34 ± 7 vs 37 ± 5, p < 0.001) were significantly lower in deceased patients than survived patients. Comparison of the laboratory parameters with the median and interquartile range values are represented in Table 3.

**Table 3.** Baseline laboratory parameters on admission in patients with COVID-19.

Parameters	Mortality		
Leukocyte count (/μl)	No	Yes	Total
IQR	3800	4620	3960
Median	6750	7020	6800
p-value	0.100		

**Lymphocyte count** (/μl)	**No**	**Yes**	**Total**
IQR	750	680	750
Median	1170	920	1140
p-value	**<0.001**		

**Neutrophil count** (/μl)	**No**	**Yes**	**Total**
IQR	3560	4410	3710
Median	4870	5725	4935
p-value	**<0.001**		

**Platelet count × 10^3^**	**No**	**Yes**	**Total**
IQR	109	100	108
Median	206	179	204
p-value	**<0.010**		

**Neutrophil/lymphocyte ratio**	**No**	**Yes**	**Total**
IQR	4.15	5.97	4.38
Median	3.89	4.31	4.15
p-value	**<0.001**		

**Platelet/lymphocyte ratio**	**No**	**Yes**	**Total**
IQR	0.127	0.157	0.129
Median	01171	0.194	0.173
p-value	**0.04**		

**C-reactive protein (mg/l)**	**No**	**Yes**	**Total**
IQR	87	102	89
Median	81	104	83
p-value	**<0.001**		

**Procalcitonin (ng/ml)**	**No**	**Yes**	**Total**
IQR	0.1	0.5	0.1
Median	0.1	0.3	0.1
p-value	**<0.001**		

**D-dimer (μg/l)**	**No**	**Yes**	**Total**
IQR	0.9	2.1	1
Median	0.8	1.3	0.8
p-value	**<0.001**		

**Ferritin (ng/ml)**	**No**	**Yes**	**Total**
IQR	500	900	533
Median	385	422	394
p-value	0.11		

**Creatinine (mg/dl)**	**No**	**Yes**	**Total**
IQR	0.4	0.8	0.4
Median	0.9	1.2	0.9
p-value	**<0.001**		

**GFR**	**No**	**Yes**	**Total**
IQR	34	49	39
Median	85	56	84
p-value	**<0.001**		

**Urea (mg/dl)**	**No**	**Yes**	**Total**
IQR	19	46	22
Median	32	51	33
p-value	**<0.001**		

**Lactate dehydrogenase (U/l)**	**No**	**Yes**	**Total**
IQR	167	226	174
Median	338	393	345
p-value	**<0.001**		

**Fibrinogen (mg/dl)**	**No**	**Yes**	**Total**
IQR	206	188	206
Median	572	561	571
p-value	0.51		

**Albumin (g/dl)**	**No**	**Yes**	**Total**
IQR	5	7	6
Median	37	34	36
p-value	**<0.001**		

**Urea/albumin ratio**	**No**	**Yes**	**Total**
IQR	0.59	1.9	0.69
Median	0.87	1.47	0.92
p-value	**<0.001**		

**Troponin (pg/ml)**	**No**	**Yes**	**Total**
IQR	4	9	5
Median	0	0	0
p-value	**<0.001**		

GFR: Glomerular filtration rate; IQR: Interquartile range.

**Figure 1.**
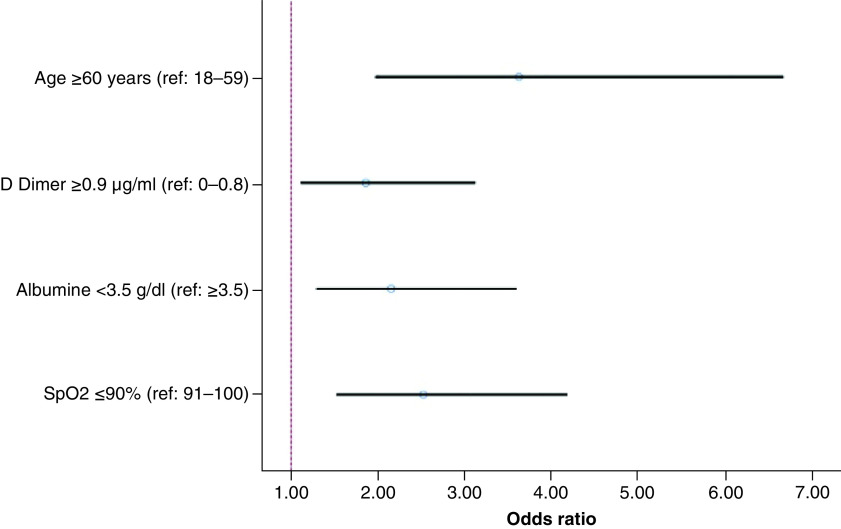
Forest plot diagram based on multivariate analysis of mortality predictors in patients with COVID-19 pneumonia.

In univariate analysis, increased age (OR = 4.63; CI = 2.93–7.32; p < 0.001), presence of dyspnea (OR = 1.55; CI = 1.02–2.35; p = 0.040) and confusion (OR = 6.18; CI = 1.89–20.57; p = 0.003), increased respiratory rate (OR = 4.06; CI = 2.42–6.84; p < 0.001), decreased SpO2 (OR = 3.50; CI = 2.38–5.15; p < 0.001), presence of any comorbidity (OR = 2.39; CI = 1.56–3.66; p < 0.001), high levels of platelet to lymphocyte ratio (OR = 1.54; CI = 1.06–2.24; p = 0.025) and D-dimer (OR = 2.66; CI = 1.73–4.09; p < 0.001), and a low level of albumin (OR = 3.63; CI = 2.32–5.69; p < 0.001) were associated with increased mortality. Multivariate analysis revealed that SpO2 (OR = 2.52; CI = 1.52–4.18; p < 0.001), albumin (OR = 2.15; CI = 1.29–3.59; p = 0.003), D-dimer (OR = 1.86; CI = 1.11–3.12; p = 0.019) and age (OR = 3.62; CI = 1.97–6.66; p < 0.001) were independent predictors for mortality (Table 4). OR and CI (95%) values for each predictor are demonstrated in the Forest plot diagram (Figure 1).

**Table 4.** Univariate and multivariate analysis of factors predicting mortality.

Logistic regression	Univariate			Multivariate		
	OR	CI	p-value	OR	CI	p-value
Age ≥60 years	4.63	2.93–7.32	**<0.001**	3.62	1.97–6.66	**<0.001**
Dyspnea	1.55	1.02–2.35	**0.040**	1.00	0.56–1.79	0.992
Confusion	6.18	1.89–20.57	**0.003**	8.05	0.72–89.84	0.090
Respiratory rate ≥28/min	4.06	2.42–6.84	**<0.001**	1.74	0.82–3.73	0.151
SpO2 ≤90%	3.50	2.38–5.15	**<0.001**	2.52	1.52–4.18	**<0.001**
Any comorbidity^†^	2.39	1.56–3.66	**<0.001**	1.31	0.72–2.37	0.373
Platelet/lymphocyte ratio ≥190	1.54	1.06–2.24	**0.025**	1.07	0.63–1.80	0.801
Albumin <3.5 g/dl	3.63	2.32–5.69	**<0.001**	2.15	1.29–3.59	**0.003**
D-dimer ≥0.9 μg/ml	2.66	1.73–4.09	**<0.001**	1.86	1.11–3.12	**0.019**

† Includes diabetes mellitus, hypertension, congestive heart failure, chronic artery disease, chronic renal failure and cerebrovascular disease.

OR: Odds ratio; SpO2: Peripheral capillary oxygen saturation.

**Figure 2.**
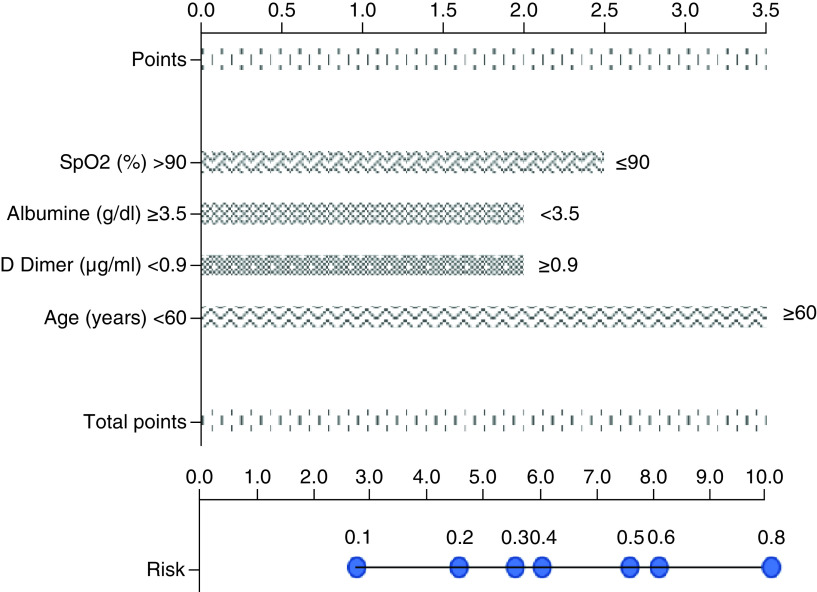
Nomogram predicting in-hospital mortality in patients with COVID-19 pneumonia.

**Figure 3.**
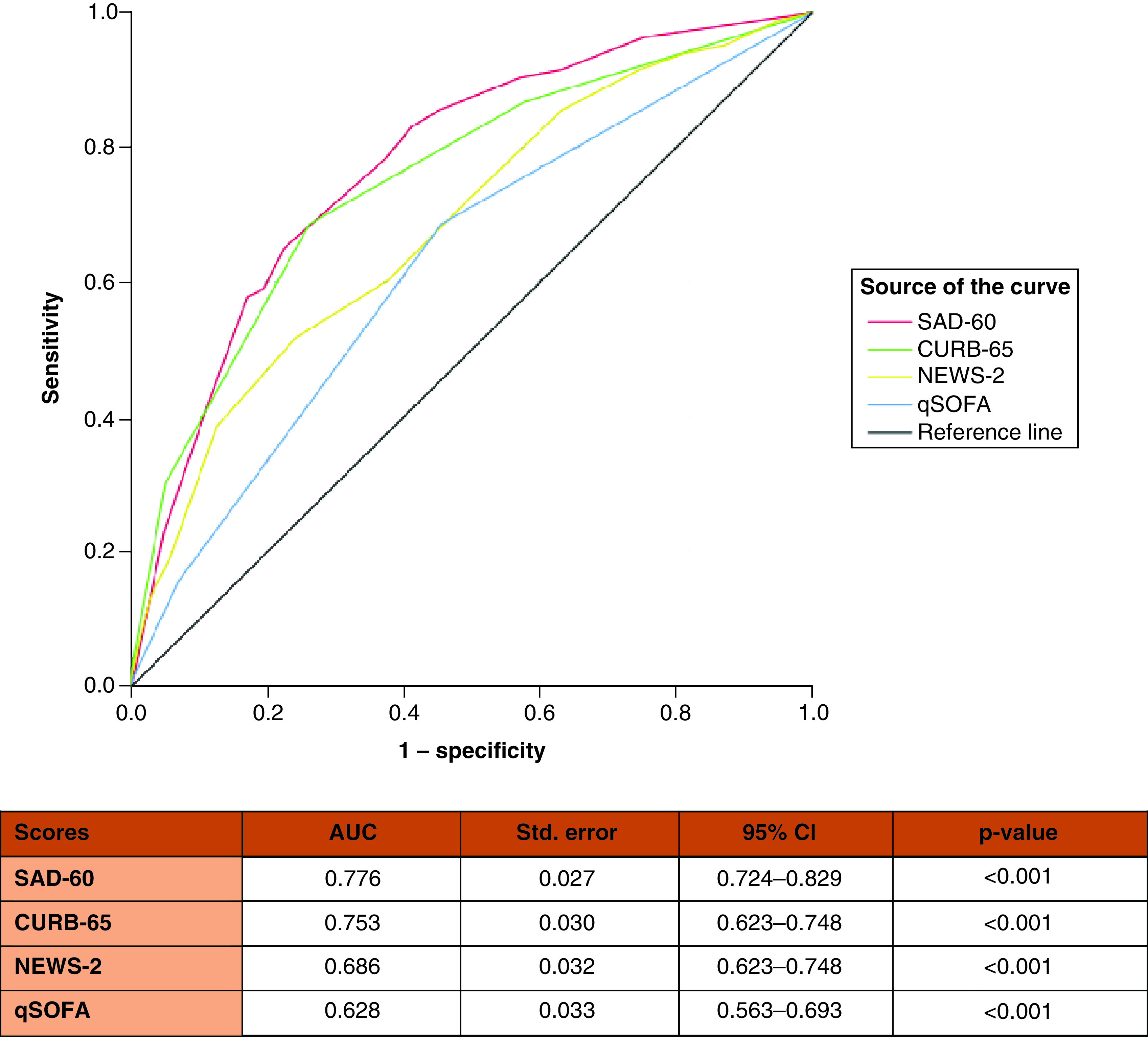
Comparison of CURB-65, qSOFA, NEWS-2 and SAD-60 for predicting mortality in hospitalized patients with COVID-19 pneumonia by receiver operating characteristic analysis.

A nomogram was developed based on four independent predictors in multivariable analysis. The score point of each parameter was determined according to its OR values. The total point, which varied from 0 to 10, was calculated by summing the points obtained from each parameter. The risk of death by total points is demonstrated in the nomogram in Figure 2. The mortality score model was given the acronym SAD-60, representing **S**pO2, **A**lbumin, **D**-dimer, age **≥60** years. The SAD-60 score (0.776) had the highest AUC compared with CURB-65 (0.753), NEWS2 (0.686) and qSOFA (0.628) scores (Figure 3). The risk of death was higher than 75% in patients with more than 8 points and lower than 25% in patients with fewer than 5.5 points.

**Figure 4.**
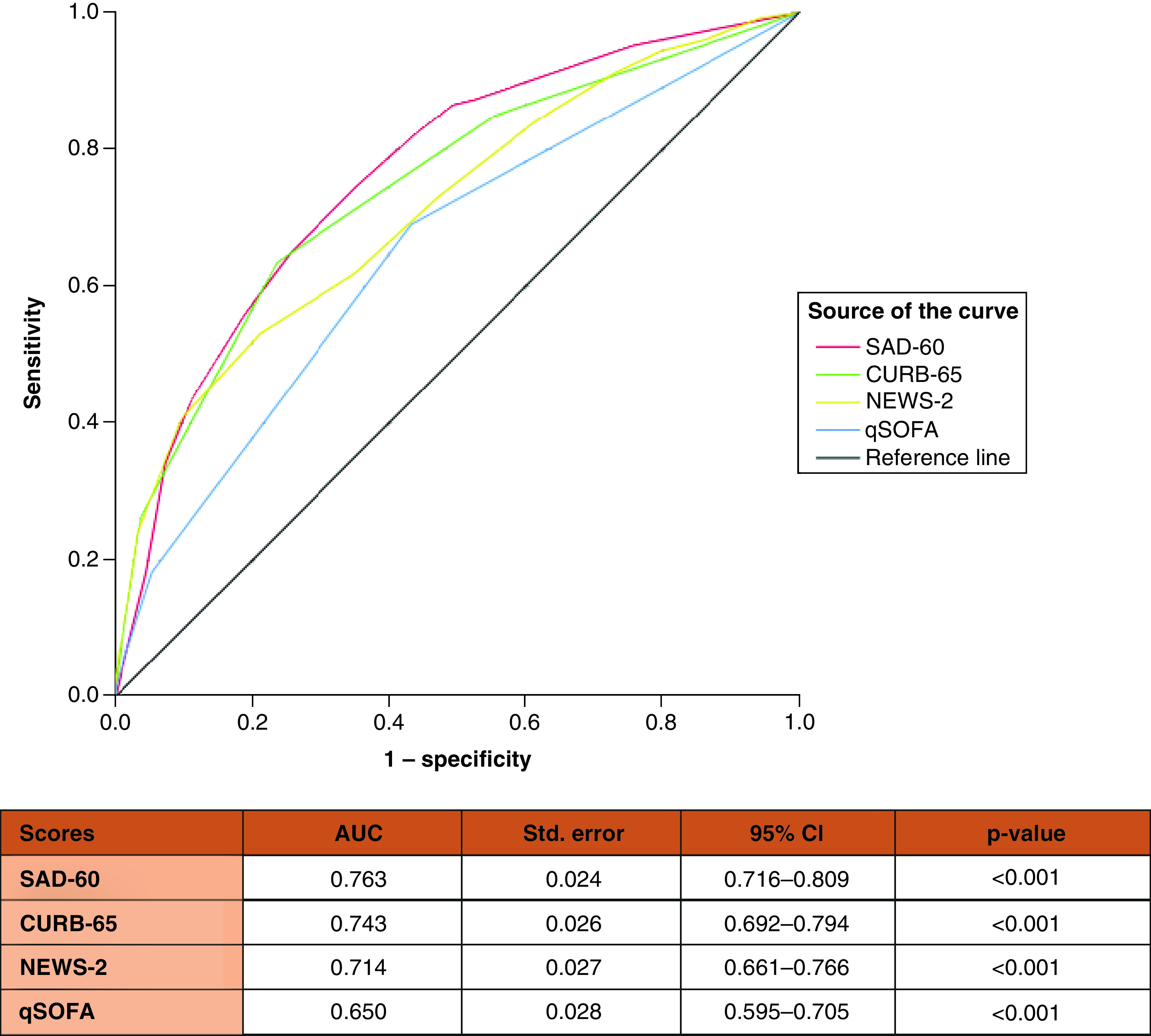
Comparison of CURB-65, qSOFA, NEWS-2 and SAD-60 for predicting intensive care admission or in-hospital death in patients with COVID-19 pneumonia by receiver operating characteristic analysis.

Additionally, the predictive ability of the SAD-60 score for ICU admission or in-hospital death was assessed and compared with CURB-65, NEWS2 and qSOFA. The results are demonstrated in Figure 4.

## Discussion

In this study, we presented a detailed analysis of 1013 patients with COVID-19 pneumonia in a multicenter retrospective cohort study and created a simple prediction model based on two biomarkers of albumin and D-dimer, as well as the clinical features of age and SpO2. The SAD-60 score that was derived from the model in the present study had a promising predictive capacity for mortality in hospitalized patients with COVID-19. Age [23], SpO2 [11], albumin [24] and D-dimer [25,26] were demonstrated as independent predictors for mortality in patients with COVID-19 in different previous studies. However, to our knowledge, this is the first study to combine these four parameters to predict mortality in hospitalized patients with COVID-19 pneumonia. In multivariate analysis, increased age, SpO2, albumin and D-dimer were associated with about 3.5-fold, 2.5-fold, twofold and twofold increased risk for mortality, respectively.

Previous studies have identified that underlying diseases are one of the risk factors for mortality [27,28]. In the present study, although underlying diseases were associated with in-hospital death, they were not detected as independent predictors in multivariate analysis.

There is an increased number of studies investigating clinical deterioration and mortality predictors of COVID-19 [27–31]. Acar *et al.* reported that 11% of COVID-19 patients (n = 75/709) died; in their study, the independent predictors of mortality were specific comorbidities, dyspnea, SpO2, hematocrit, CRP, aspartate aminotransferase and ferritin. They developed a novel score with the combination of these seven predictors in addition to age. In the study of Guner *et al.*, 15.2% of COVID-19 patients (n = 104/686) transferred to the ICU [29]. In their final model, the independent predictors of the need for ICU transfer were SpO2, CRP, procalcitonin, lactate dehidyrogenase and troponin. Guner *et al.* reported a good predictive value in the ROC analysis (AUC = 0.93; CI = 0.90–0.95) [29]. Bayram *et al.* [30] developed a novel score named *CAPA*, which allows for the prediction of mortality and ICU admission in patients with COVID-19. They reported that the AUC values of the CAPA score in predicting mortality and ICU admission were 0.67 and 0.66, respectively. In our study, the AUC values of the SAD-60 score in predicting mortality and ICU admission were 0.776 and 0.763, respectively. However, these studies were conducted in single tertiary care centers. Liang *et al.* [31] established the COVID-GRAM score with a cohort of 1590 COVID-19 patients from 575 hospitals in China and demonstrated that the mean AUC was 0.88 (CI = 0.85–0.91) in the development cohort.

Although most patients have mild or moderate disease, COVID-19 can progress to severe disease and result in acute respiratory distress syndrome, multiorgan failure, septic shock and death [2]. Therefore, early stratifying of COVID-19 patients based on disease severity is vital. CURB-65 has been the widely used scoring system for severity classification, outcome and mortality prediction of community-acquired pneumonia. NEWS2 has been recommended by the National Institute of Clinical Excellence (NICE) for the prediction of clinical deterioration in patients with COVID-19 [32]. However, although there are studies to identify risk factors for disease progression and to develop scoring models in patients with COVID-19, no concensus has been reached [18,33–35]. In addition, previous reports do not identify cut-off values of continues variables [36] or have not used combined parameters similar to our previous report (blinded). Even if studies categorize continuous variables, they do not stratify patients according to mortality risk by scoring [37,38]. These issues cause difficulties in calculation of the risk by physicians. In the present study, a simplified nomogram was used to assess the risk of mortality in patients with COVID-19 pneumonia.

The SAD-60 score, which was derived from the model in the present study, could be helpful for detecting patients at high risk of clinical deterioration and mortality. This might improve patient outcomes by enhancing physicians clinical decision making. Indicators of inflammation could be useful for predicting prognosis in patients with COVID-19. Our biomarker-based model incorporated albumin and D-dimer. Recent studies have confirmed that these biomarkers provide substantive information about clinical deterioration and the risk of mortality [25,26,38–40]. In addition, some studies revealed the role of albumin in COVID-19 prognostication reflecting both possible liver damage, inflammation and the nutritional status of patients [41,42].

This study has several strengths. First, this was a multicenter study conducted in six hospitals with 1013 patients with COVID-19. Second, different types of variables, such as multiple comorbidities, symptoms, vital signs and laboratory parameters, were included in the multivariate regression analysis. Moreover, as laboratory parameters were routinely tested in all six hospitals, we could collect biomarkers probably associated with disease severity. Third, we had a relatively a large sample size.

This study has several limitations. First, our study was retrospectively conducted. Second, external validation was not performed. The generalizability of the results might be limited even with this being a multicenter study. Therefore, we need new large-scale studies to further improve the robustness of this model. Last, we did not perform longitudinal evaluation of vital signs and laboratory parameters.

A part of this study was presented at IDWeek-2021. The abstract, Table 4 & Figure 3 were published in the journal *Open Forum Infectious Diseases* [43].

## Conclusion

We created a simple prediction model based on two biomarkers, as well as the clinical features of age and SpO2, and demonstrated that the SAD-60 score has promising predictive capacity for mortality in hospitalized patients with COVID-19. Thus, patients with high risk scores at admission should be carefully monitored and preventive strategies should be implemented to reduce mortality.

**Summary points**

- The pandemic of COVID-19 continues to be a significant public health issue.
- In this retrospective multicenter study, the aim was to explore a novel risk score to predict mortality in hospitalized patients with COVID-19 pneumonia. In addition, the accuracy of the novel risk score with CURB-65, qSOFA and NEWS2 scores was compared.
- A total of 1013 patients with COVID-19 were included. In-hospital death occurred in 124 (12.2%) patients.
- Multivariate analysis revealed that peripheral capillary oxygen saturation, albumin, D-dimer and age were independent predictors for mortality.
- The mortality score model was given the acronym SAD-60, representing **S**pO2, **A**lbumin, **D**-dimer, age **≥60** years.
- The SAD-60 score (0.776) had the highest area under the curve compared with CURB-65 (0.753), NEWS2 (0.686) and qSOFA (0.628) scores.
- The SAD-60 score has a promising predictive capacity for mortality in hospitalized patients with COVID-19.
